# Cerebral ischemia initiates an immediate innate immune response in neonates during cardiac surgery

**DOI:** 10.1186/1742-2094-10-24

**Published:** 2013-02-07

**Authors:** Selma O Algra, Kathelijne M Groeneveld, Alvin WL Schadenberg, Felix Haas, Fabiola CM Evens, Jenny Meerding, Leo Koenderman, Nicolaas JG Jansen, Berent J Prakken

**Affiliations:** 1 Department of Pediatric Cardiothoracic Surgery, Wilhelmina Children’s Hospital, University Medical Center Utrecht, Lundlaan 6, 3584, Utrecht, EA, The Netherlands; 2 Department of Surgery, University Medical Center Utrecht, Heidelberglaan 100, 3584, Utrecht, CX, The Netherlands; 3 Department of Pediatric Immunology, Center for Cellular and Molecular Intervention, University Medical Center Utrecht, Lundlaan 6, 3584, Utrecht, EA, The Netherlands; 4 Pediatric Intensive Care Unit, Wilhelmina Children’s Hospital, University Medical Center Utrecht, Lundlaan 6, 3584, Utrecht, EA, The Netherlands; 5 Pediatric Cardiothoracic Surgery, University Medical Center Utrecht, Utrecht, the Netherlands

**Keywords:** Cardiac surgery, Cerebral blood flow, Hypothermia, Inflammation, Neonatal ischemia, Randomized controlled trials

## Abstract

**Background:**

A robust inflammatory response occurs in the hours and days following cerebral ischemia. However, little is known about the immediate innate immune response in the first minutes after an ischemic insult in humans. We utilized the use of circulatory arrest during cardiac surgery to assess this.

**Methods:**

Twelve neonates diagnosed with an aortic arch obstruction underwent cardiac surgery with cardiopulmonary bypass and approximately 30 minutes of deep hypothermic circulatory arrest (DHCA, representing cerebral ischemia). Blood samples were drawn from the vena cava superior immediately after DHCA and at various other time points from preoperatively to 24 hours after surgery. The innate immune response was assessed by neutrophil and monocyte count and phenotype using FACS, and concentrations of cytokines IL-1β, IL-6, IL-8, IL-10, TNFα, sVCAM-1 and MCP-1 were assessed using multiplex immunoassay. Results were compared to a simultaneously drawn sample from the arterial cannula. Twelve other neonates were randomly allocated to undergo the same procedure but with continuous antegrade cerebral perfusion (ACP).

**Results:**

Immediately after cerebral ischemia (DHCA), neutrophil and monocyte counts were higher in venous blood than arterial (*P* = 0.03 and *P* = 0.02 respectively). The phenotypes of these cells showed an activated state (both *P* <0.01). Most striking was the increase in the ‘non-classical’ monocyte subpopulations (CD16^intermediate^; arterial 6.6% vs. venous 14%; CD16+ 13% vs. 22%, both *P* <0.01). Also, higher IL-6 and lower sVCAM-1 concentrations were found in venous blood (both *P* = 0.03). In contrast, in the ACP group, all inflammatory parameters remained stable.

**Conclusions:**

In neonates, approximately 30 minutes of cerebral ischemia during deep hypothermia elicits an immediate innate immune response, especially of the monocyte compartment. This phenomenon may hold important clues for the understanding of the inflammatory response to stroke and its potentially detrimental consequences.

**Trial registration:**

ClinicalTrial.gov: NCT01032876

## Background

It is well established that cerebral ischemia induces an immune response on many different levels. Both in the parenchyma and the systemic circulation, within hours after the insult, cytokines are produced in vast amounts and leukocytes are activated and migrate into the injured brain [1-5]. The various immune cells and their subpopulations have very different effects on the lesion and thus the outcome for the patient [6].

The exact mechanisms in the pathway from the cessation of blood flow to the establishment of stroke and its outcome have predominantly been studied in animal models. These have shown the infiltration of neutrophils and monocytes into the cerebral tissue starting from 4 to 6 hours after the ischemic insult, with these cells showing phenotypical changes in the periphery for weeks following the event [2,3]. In adults with a cerebrovascular accident or a transient ischemic attack, it is the systemic compartment which has been studied most. Proinflammatory cytokine concentrations have been associated with poor neurological outcome after stroke, as have innate immune cell numbers and phenotypes [7-9]. For example, Smith *et al.* have found high TNFα and activated neutrophils to be correlated to worse symptoms after stroke [9]. Others have shown that monocytes are highly associated to prognosis, with the different monocyte subpopulations having variable effects, likely due to their specific characteristics and functions [4]. For example, the abundance of ‘classical’ (CD16-) monocytes in the peripheral circulation following stroke were shown to be associated with a worse outcome [8]. Data on the immune response to cerebral ischemia in neonates is scarce. There have been studies on the effects of perinatal asphyxia, which show a burst in cytokines and activation of neutrophils and monocytes within 24 hours, similar to the response in adults [10-15].

Although many studies have characterized the immune response to stroke in the hours and days after the insult, the immediate effect of cerebral ischemia has never been assessed. The unique opportunity to assess this arises in the field of cardiac surgery, where procedures performed with the use of cardiopulmonary bypass (CPB) may include a period of global circulatory arrest at a deep hypothermic temperature (‘deep hypothermic circulatory arrest’, DHCA). This allows for the study of the immediate response to approximately 30 minutes of induced cerebral ischemia. We assessed the inflammation in the cerebral circulation by characterization of neutrophils and monocytes and measurement of cytokines IL-1β, IL-6, IL-8, IL-10, TNFα, sVCAM-1 and MCP-1, as these markers are known to respond within hours after cerebral ischemia [1,3,7,8,10-14,16-20]. The results were compared to those from patients undergoing the same surgical procedure at the same temperature, but with continuous cerebral perfusion, and thus without any apparent ischemia (‘antegrade cerebral perfusion’, ACP). We hypothesized that DHCA would have an immediate ‘proinflammatory’ effect on innate immune cells and cytokines, whereas ACP would not affect these markers of systemic inflammation.

## Methods

### Patients and surgical procedures

Neonates presenting with a hypoplastic aortic arch between 2009 and 2011 were assigned to undergo aortic arch reconstruction. This procedure entails the use of either DHCA or ACP during CPB. The current study was performed as part of a prospective randomized controlled trial comparing DHCA and ACP in terms of neurological outcome (clinicaltrials.gov number NCT01032876). In the subgroup reported here, data on cerebral inflammatory markers were collected. Surgery and CPB was performed as previously described [21]. The institutional medical ethics committee approved the study and all parents gave informed consent for enrollment.

### Sample collection

**Figure 1:**
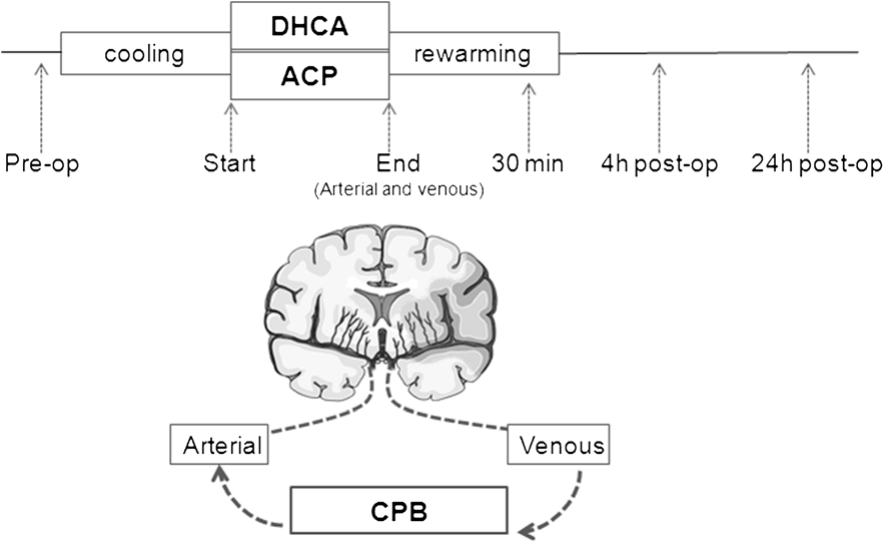
**Sample collection during CPB.** Upper picture shows all time points at which blood samples were collected. The first was before CPB (‘Pre-op’). Once on CPB, patients were cooled to deep hypothermia and DHCA or ACP was initiated (‘Start’). At the end of DHCA or ACP (‘End’), full-flow CPB was recommenced and the patient was rewarmed to normothermia, during which the ‘30 min’ sample was drawn. Four and 24 hours after surgery, the final samples were collected (‘4 h post-operative’ and ‘24 h post-operative’). The lower picture depicts the arterial and venous samples drawn at the ‘End’ time point. Samples were simultaneously drawn from the arterial and venous cannulae, transporting blood to and from the brain, respectively. ACP, antegrade cerebral perfusion; CPB, cardiopulmonary bypass; DHCA, deep hypothermic circulatory arrest.

Blood samples were drawn at various time points (see Figure 1); 1) ‘preoperative’: before the start of CPB and after the administration of dexamethasone 1 mg/kg to the patient; 2) ‘Start of DHCA/ACP’: after cooling, at deep hypothermia on full-flow CPB, just before the start of either DHCA or ACP; 3) ‘End’: immediately (0 to 3 minutes) following DHCA or ACP; 4) ‘30 min reperfusion’: 30 minutes after recommencement of full body CPB (during rewarming); 5) ‘4 h postoperative’: 4 hours after the end of DHCA or ACP, with the patient off CPB and on the intensive care unit; 6) ‘24 h postoperative’: 24 hours after surgery.

**Figure 2:**
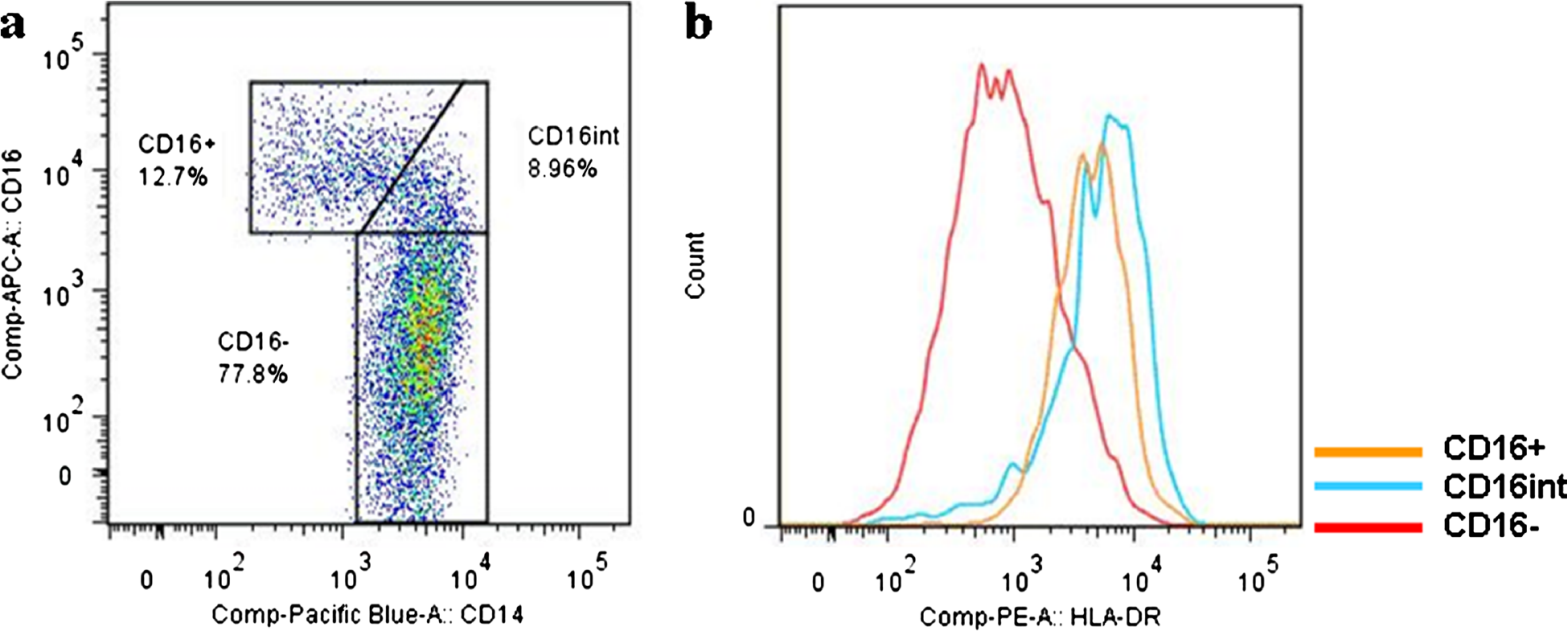
**Monocyte subpopulations.** (**a**) Gating of monocytes into CD16+, CD16int (intermediates) and CD16+ subpopulations. (**b**) CD16- monocytes show a low expression of HLA-DR, whereas CD16int and CD16+ show a high expression (MFIs of 663 (IQR 337 to 867), 2174 (1189 to 4884) and 3903 (1372 to 8393), respectively). IQR, interquartile range; HLA-DR, human leukocyte antigen D-related; MFI, median fluorescence intensity.

At the ‘Start DHCA/ACP’ time point (at deep hypothermia, on full-flow CPB), leukocyte counts are lower than before surgery, due to the expansion of the circulating volume after connection to the CPB circuit. For better comparison with preoperative leukocyte counts, the count during CPB at ‘Start’ was corrected for this dilution, based on the volume of the CPB circuit. For example, a child of 3 kg is expected to have 270 ml of circulating volume preoperatively, and when a CPB circuit volume of 810 ml is added, the total circulating volume makes for a total volume of 1080 ml, hence a 4-times dilution. The leukocyte count at ‘Start’ can then be corrected for dilution by being multiplied by 4 to attain the cell count corrected for dilution. The same applies to the ‘End’ and ‘30 min’ time points. We depicted these corrected counts in grey in Figure 2a and b.

At the ‘End’ time point, blood samples were drawn from two different locations of the CPB circuit, named the ‘arterial’ and ‘venous’ samples (see Figure 1). The arterial sample represents blood going to the brain, whereas the venous sample represents blood exiting the brain. The arterial sample was drawn from the arterial line, which connects the CPB machine to the aorta of the patient. The venous sample was drawn from the venous cannula, which is situated in the vena cava superior of the patient, where the venae azygos and anonyma were temporarily occluded in order to obtain blood specifically coming from the brain, and the first 10 ml of blood was discarded as it approximates the dead space of the cannula.

### Laboratory analyses

Blood samples were kept on ice during all handling and staining procedures. Plasma samples were analyzed by multiplex immunoassay to measure levels of IL-1β, IL-6, IL-8, IL-10, TNFα, sVCAM-1 and MCP-1 with the Bio-Plex™ suspension array system (Bio-Rad Laboratories, Hercules, CA, USA) as previously described [22]. Neutrophil and monocyte counts were performed by manual microscopy; hematocrit and thrombocyte counts were analyzed by an automated cell counter.

**Figure 3:**
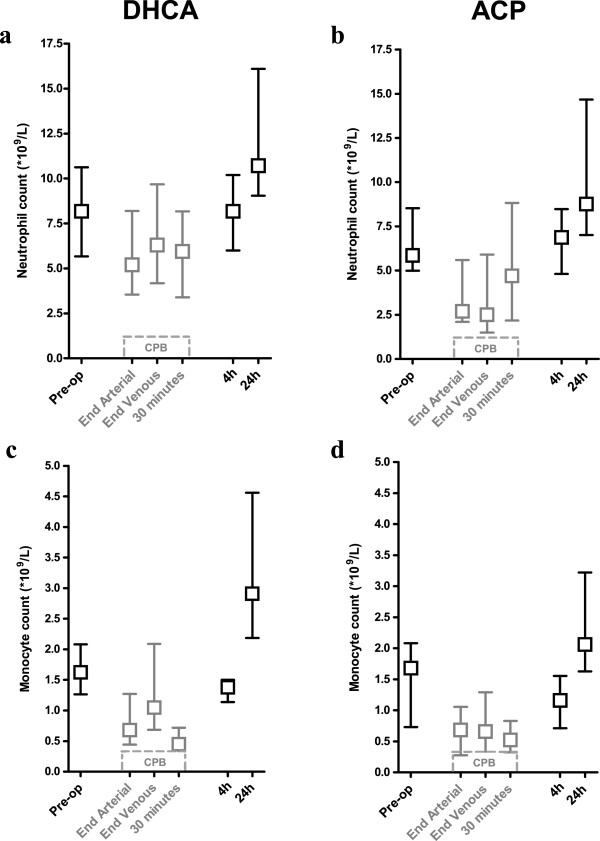
**Deep hypothermic CPB decreases neutrophil and monocyte counts.** Perioperative time course of neutrophil and monocyte and counts, in DHCA and ACP groups separately (DHCA; (**a**) and (**c**); ACP; (**b**) and (**d**)). During CPB, counts were corrected for the dilution of the CPB circuit (shown in grey). ACP, antegrade cerebral perfusion; CPB, cardiopulmonary bypass; DHCA, deep hypothermic circulatory arrest.

As shown in Figure 3, monocytes were categorized into three monocyte subpopulations (CD16-, CD16^intermediate^ (CD16int), and CD16) as proposed by Ziegler-Heitbrock *et al.*[23,24]. For neutrophil phenotyping, fluorescently labeled monoclonal antibodies (mAb) directed against CD11b (RPE, clone 2LPM19c; Dako, Glostrup, Denmark) and CD62L (FITC, clone Dreg56; BD Biosciences, Franklin Lakes, New Jersey, USA) were used for flow cytometry. For monocyte phenotyping, CD14 (Pacific Blue, 301815 clone M5E2; BioLegend, San Diego, CA, USA), HLA-DR (PE, 347401; BD Biosciences), CD16 (APC, MHCD1605, clone 3 G8, CalTag Medsystems, Buckingham, UK) and CD62L (FITC, 555543, clone Dreg 56; BD Biosciences), The FACSCanto flow cytometer (BD Biosciences) was used to record cells and FacsDiva software (BD Biosciences), Facs Express (De Novo software, Thornhill, ON, Canada) and FlowJo (Ashland, OR, USA) software were used for analysis. Neutrophils were identified according to their specific forward- and sideward-scatter signals. Expressions of CD11b and CD62L were calculated as activational markers (with CD11b increasing, and CD62L decreasing during activation).

### Statistical analyses

Values in graphs and text are stated as numbers (% of population) or medians (interquartile range (IQR)). Differences between arterial and venous samples were tested using Wilcoxon signed-rank test. Differences between DHCA and ACP for time points from ‘30 min reperfusion’ onward were tested by Mann–Whitney *U* test. IBM SPSS Statistics version 19 (IBM Corp., Armonk, NY, USA) was used for statistical calculations and Prism version 5.03 was used to depict graphs (GraphPad Software, San Diego, CA, USA).

## Results

### Baseline characteristics

Data were collected from 24 neonates (n = 12 for DHCA, n = 12 for ACP). All neonates underwent aortic arch reconstruction as well as intracardiac procedures for specific diagnoses listed in Table 1. Surgical and CPB data were similar at the start of DHCA or ACP. Also, inflammatory profiles were comparable preoperatively and at the start of either DHCA or ACP, as shown in Table 2.

**Table 1:** Patient characteristics

	**DHCA (n = 12)**	**ACP (n = 12)**
Male sex	8/12 (67)	9/12 (75)
Age at surgery (days)	9 (8–11)	12 (8–15)
Diagnosis:		
Hypoplastic left/right heart syndrome or complex	8 (67)	5 (42)
Aortic arch interruption/coarctation	3 (25)	5 (42)
Double outlet right ventricle or double inlet left ventricle	1 (8)	2 (17)
Duration of cooling (minutes)	33 (20–43)	30 (28–36)
Nasal temperature at deep hypothermia (°C)	17.7 (17.1–18.1)	17.5 (16.6–17.9)
Duration of DHCA or ACP (minutes)	33 (23–38)	44 (33–53)
Dilution factor circulating volume during CPB	3.8 (2.3–4.5)	4.2 (2.6–4.6)

Values represent number (% of group) or medians (interquartile ranges). *ACP*, antegrade cerebral perfusion; *CPB*, cardiopulmonary bypass; *DHCA*, deep hypothermic circulatory arrest.

**Table 2:** Baseline inflammatory characteristics

**Neutrophils and monocytes**	**DHCA**	**ACP**
**Preoperative**		
Monocyte count	1.6 (1.3–2.1)	1.9 (1.1–2.1)
% CD16- monocytes	83 (71–91)	74 (52–83)
% CD16int monocytes	7.0 (5.0-14)	12 (6.7–22)
% CD16+ monocytes	7.0 (4.5–15)	10 (5.5–22)
Neutrophil count	8.2 (5.7–10.6)	6.0 (5.4–8.8)
Neutrophil CD62L expression	166 (103–257)	294 (221–329)
Neutrophil CD11b expression	426 (264–717)	523 (265–685)
**Start DHCA or ACP**		
Monocyte count	0.17 (0.09–0.32)	0.20 (0.13–0.27)
% CD16- monocytes	81 (70–86)	78 (66–85)
% CD16int monocytes	6.5 (3.5–9.0)	6.6 (3.7–8.6)
% CD16+ monocytes	14 (7.4–19)	14 (7.3–23)
Neutrophil count	1.4 (0.8–2.2)	1.0 (0.5–1.4)
Neutrophil CD62L expression	189 (104–262)	205 (146–288)
Neutrophil CD11b expression	519 (322–743)	673 (468–792)
**Cytokines and chemokines**		
**Start DHCA or ACP**		
IL-1β	0.18 (0.00-1.1)	0.18 (0.00-0.62)
IL-6	18 (1.0–21)	8.1 (2.3–16)
IL-8	7.2 (5.6–35)	5.3 (3.0–10)
IL-10	42 (9.7–61)	12 (6.3–32)
**Values corrected for dilution**		
IL-1β	0.82 (0.72–18)	0.81 (0.78–12)
IL-6	59 (15–103)	36 (8.1–65)
IL-8	28 (18–67)	26 (11–65)
IL-10	105 (42–337)	74 (23–135)

Values represent medians (interquartile ranges). Neutrophil and monocyte counts in 10^9^/L, neutrophil expressions in median fluorescence intensity (MFI), cytokine and chemokine concentrations in pg/ml. *ACP*, antegrade cerebral perfusion; *DHCA*, deep hypothermic circulatory arrest.

### Deep hypothermic CPB decreases neutrophil and monocyte counts

Cell numbers were available for all 24 neonates (n = 12 for both groups). Figure 2 shows the perioperative change in neutrophil and monocyte counts for the DHCA and ACP groups separately. In grey, the counts during deep hypothermic, full-flow CPB are depicted, corrected for the dilution of the CPB circuit. At 4 hours after surgery, neutrophil and monocyte numbers are back to their preoperative values and rise further at 24 hours.

### Cerebral ischemia increases neutrophils and monocyte counts

**Figure 4:**
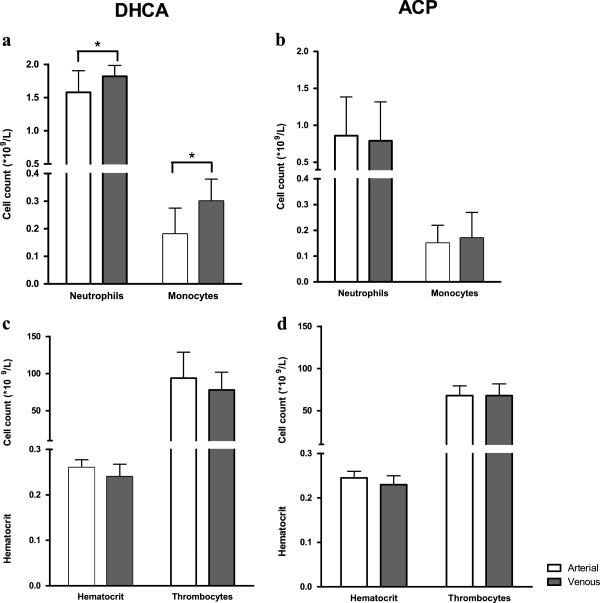
**Cerebral ischemia increases neutrophils and monocyte counts.** (**a**) Immediately after cerebral ischemia (i.e., DHCA), neutrophil and monocyte counts are increased in the venous sample compared to arterial (*P* = 0.03 and *P* = 0.02, respectively). (**b**) In continuous cerebral perfusion (i.e., ACP) there is no difference in neutrophil and monocyte counts. (**c**) To rule out the effect of hyperviscosity in the venous sample after cerebral ischemia, hematocrit and thrombocyte numbers were assessed revealing no difference after DHCA. (**d**) Also after ACP there is no difference in hematocrit and thrombocyte number. ACP, antegrade cerebral perfusion; DHCA, deep hypothermic circulatory arrest.

To assess the direct effect of cerebral ischemia on leukocyte concentrations, we compared the absolute cell counts of the cerebral venous sample to the arterial sample, both obtained immediately after DHCA. Results are depicted in Figure 4a, which shows significantly increased neutrophil numbers in venous blood compared to arterial (median arterial 1.6 × 10^9^/L (IQR 1.0 to 2.2) vs. venous 1.9 × 10^9^/L (1.4 to 2.1); *P* = 0.03). Monocyte counts also showed an increase from arterial to venous (median arterial 0.18 × 10^9^/L (0.09 to 0.28) vs. venous 0.30 × 10^9^/L (0.21 to 0.38), *P* = 0.02). In neonates with continuous cerebral perfusion (ACP), leukocyte numbers did not change (neutrophils *P* = 0.72 and monocytes *P* = 0.40; Figure 4b).

To rule out an effect of hyperviscosity on the increased leukocyte number after cerebral ischemia, hematocrit and thrombocyte numbers were compared in arterial and venous blood. As shown in Figure 4c and d, these were stable after both DHCA and ACP (DHCA: hematocrit *P* = 0.07 and thrombocytes *P* = 0.15; ACP: hematocrit *P* = 0.44 and thrombocytes *P* = 0.96).

### Cerebral ischemia leads to an immediate activation of neutrophils and monocytes

Next, we questioned whether neutrophils exiting the cerebral circulation after ischemia were not only more abundant, but also more activated.

**Figure 5:**
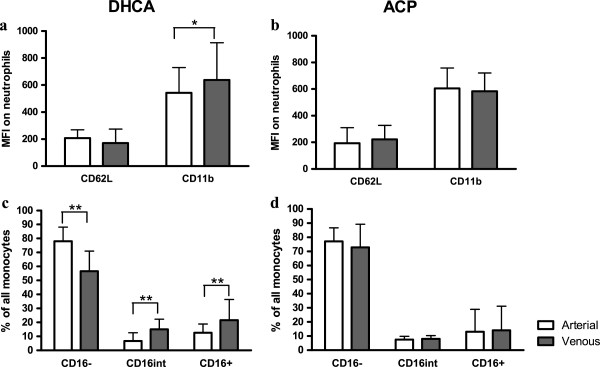
**Cerebral ischemia leads to an immediate activation of neutrophils and monocytes.** (**a**) After cerebral ischemia (i.e., DHCA), CD62L expression is the same in arterial and venous blood, but CD11b expression is higher in the venous sample compared to arterial (*P* <0.01). (**b**) After continuous cerebral perfusion (i.e., ACP), neutrophil CD11b and CD62L expressions remain stable. (**c**) After cerebral ischemia (DHCA), in the venous sample, the proportion of monocytes that is CD16- is decreased compared to arterial, due to an increase in CD16intermediate and CD16+ monocyte subpopulations (all *P* <0.01). (**d**) After continuous cerebral perfusion (ACP), the monocyte subpopulations remain unchanged.

To this end, we assessed neutrophil phenotype using CD62L (which is shed during activation) and CD11b (which increases during activation). As shown in Figure 5a, after cerebral ischemia (i.e., DHCA), although CD62L did not change (*P* = 0.48), CD11b did show a small, but significant increase from arterial to venous (arterial median fluorescence intensity (MFI) 341 (286 to 543), venous 382 (313 to 638); *P* <0.01). In continuous cerebral perfusion (i.e., ACP), no changes in expression of CD62L and CD11b were observed (CD62L *P* = 0.06, CD11bb *P* = 0.26; see Figure 5b).

Furthermore, we assessed changes in the composition of the monocyte compartment after cerebral ischemia. As depicted in Figure 5c and d, after cerebral ischemia (i.e.,, DHCA), the percentage of CD16-negative monocytes decreased significantly in the venous sample compared to arterial (median arterial 78% (IQR 67 to 88), venous 57% (45 to 71), *P* <0.01; see Figure 5c). In contrast, the CD16^intermediate^ and CD16+ populations increased from arterial to venous, reflecting increased monocyte activation (CD16^intermediate^, median arterial 6.6% (4.5 to 8.7), venous 14 (11 to 17); CD16+ median arterial 13% (IQR 8.6 to 19), venous 22% (17 to 36); both *P* <0.01). After ACP, monocyte subpopulations remained unchanged (CD16-negative *P* = 0.57, CD16^intermediate^*P* = 0.48, CD16+: *P* = 0.51; see Figure 5d).

### Cerebral ischemia increases IL-6, and decreases sVCAM-1 concentrations

**Figure 6:**
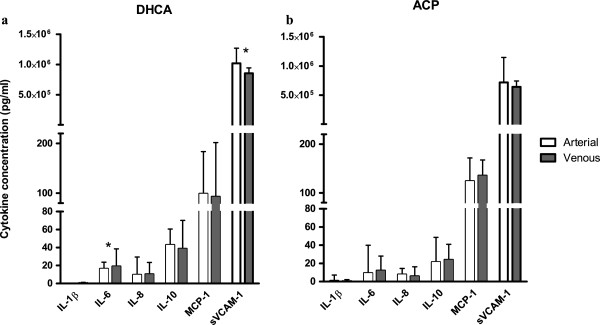
**Cerebral ischemia increases IL-6, and decreases sVCAM-1 concentrations.** (**a**) After cerebral ischemia (i.e., DHCA), serum IL-6 is mildly increased, and sVCAM-1 decreased, in the venous sample compared to arterial (both *P* = 0.03). (**b**) After continuous cerebral perfusion (ACP), all cytokine levels remain stable.

To assess whether the proinflammatory profile of the cells was also reflected in the soluble compartment, cytokines and chemokines were measured in the first 18 neonates included in this study (n = 10 DHCA, n = 8 ACP). Figure 6 shows arterial and venous concentrations after DHCA and ACP. After cerebral ischemia (i.e., DHCA), IL-6 levels significantly increased from arterial to venous (median arterial 16 pg/ml (0.6 to 22); venous 19 pg/ml (1.1 to 39); *P* = 0.03).

Furthermore, soluble VCAM-1 significantly decreased in this group (median arterial 1.0 × 10^6^ pg/ml (0.83 to 1.3 × 10^6^); vs. venous 0.8 × 10^6^ pg/ml (0.8 to 9.4 × 10^6^), *P* = 0.03). These cytokines did not show any change in concentration in the ACP group (IL-6 *P* = 0.49; sVCAM-1 *P* = 0.26). Regarding the other cytokines IL-1β, IL-8, IL-10 and MCP-1, these did not change significantly from arterial to venous in either the DHCA or ACP group (DHCA, IL-1β *P* = 0.08, IL-8 *P*= 0.17, IL-10 *P*= 0.31, MCP *P* = 0.37; ACP, IL-1β *P* = 0.48, IL-8 *P* = 0.16, IL-10 *P* = 0.16, MCP *P* = 0.48). TNFα was below the detection threshold in all patients.

### Differences between DHCA and ACP subside at later time points

As listed in Table 3, after 30 minutes of reperfusion, there were no differences to be found in neutrophil and monocyte count or phenotype between DHCA and ACP. The same applies to 4 hours postoperatively, although at 24 hours there was a slight difference in monocyte subpopulations, with less CD16+ monocytes in the DHCA group (*P* = 0.01).

**Table 3:** Inflammatory results for later time points

**Neutrophils and monocytes**	**DHCA**	**ACP**	***P***
**30 min reperfusion**			
Monocyte count	0.13 (0.06-0.23)	0.12 (0.07-0.14)	0.76
% CD16- monocytes	68 (60–85)	74 (50–88)	0.84
% CD16int monocytes	7.4 (5.7-14)	6.1 (2.4-10)	0.36
% CD16+ monocytes	19 (9.2-33)	16 (6.0-43)	0.76
Neutrophil count	1.5 (1.2-1.9)	1.1 (0.7-1.7)	0.24
Neutrophil CD62L expression	193 (67–227)	187 (134–307)	0.44
Neutrophil CD11b expression	610 (469–816)	672 (508–926)	0.85
**4 h postoperative**			
Monocyte count	3.1 (1.7-4.8)	2.0 (1.5-2.4)	0.15
% CD16- monocytes	86 (85–95)	87 (78–94)	0.74
% CD16int monocytes	6.8 (4.9-8.7)	7.5 (3.2-11)	0.72
% CD16+ monocytes	6.0 (1.7-8.5)	4.5 (2.4-9.9)	0.88
Neutrophil count	8.2 (6.0-10.2)	6.9 (4.8-8.5)	0.43
Neutrophil CD62L expression	281 (253–300)	292 (257–356)	0.43
Neutrophil CD11b expression	478 (276–621)	462 (237–594)	0.65
**24 h postoperative**			
Monocyte count	1.4 (1.0-2.2)	0.9 (0.7-1.3)	0.29
% CD16- monocytes	88 (78–94)	81 (73–91)	0.23
% CD16int monocytes	10 (6.0-21)	10 (6.0-26)	0.61
% CD16+ monocytes	2.9 (1.7-3.0)	6.0 (3.0-7.0)	0.01
Neutrophil count	11 (9.0-16)	8.8 (7.0-15)	0.32
Neutrophil CD62L expression	260 (196–322)	300 (227–336)	0.35
Neutrophil CD11b expression	359 (227–491)	288 (176–368)	0.22
**Cytokines and chemokines**			
**4 h postoperative**			
IL-1β	0.25 (0.25-0.25)	0.25 (0.25-4.2)	0.61
IL-6	91 (48–114)	49 (41–65)	0.14
IL-8	77 (59–109)	53 (29–64)	0.04
IL-10	2760 (2474–7077)	3112 (768–5758)	0.42
**24 h postoperative**			
IL-1β	0.25 (0.00-3.9)	2.2 (0.51-3.5)	0.48
IL-6	19 (11–34)	23 (7.6-33)	0.76
IL-8	44 (27–67)	15 (11–37)	0.05
IL-10	24 (16–33)	15 (7.4-31)	0.20

Values represent medians (interquartile ranges). Neutrophil and monocyte counts in 10^9^/L, neutrophil expressions in median fluorescence intensity (MFI), cytokine and chemokine concentrations in pg/ml. *ACP*, antegrade cerebral perfusion; *DHCA*, deep hypothermic circulatory arrest.

Regarding cytokines and chemokines, absolute concentrations were much higher after surgery than during surgery, but when the intraoperative values are corrected for dilution, IL-6 values remain approximately stable. When comparing the two groups, only at 4 hours postoperatively was there a higher IL-8 concentration in the DHCA group (*P* = 0.04), other cytokines were not significantly different.

## Discussion

In the present study, we observed that in neonates undergoing cardiac surgery, cerebral ischemia during deep hypothermia elicited an immediate inflammatory response in the cerebral circulation. Not only were there significant changes in the soluble milieu; neutrophils and monocytes acquired an activated phenotype and were even increased in number upon leaving the cerebral circulation. This was not the case in the situation where the brain was continuously perfused. This phenomenon has not been reported on previously and may hold important clues for further research on the inflammatory response to stroke.

Previous studies involving cerebral ischemia and inflammation, both in neonates and adults, have found changes in peripheral immune cells within hours after the ischemic insult [1,3,8,10-14,16]. In the same time frame, leukocytes are reported to start their migration into the ischemic lesion [2-4]. However, to our knowledge, the immediate effect of ischemia on the cerebral circulation has not been assessed previously. There have been reports of other models of ischemia in other organ systems, for example in limb ischemia and reperfusion during orthopedic surgery. These have found neutrophils and monocytes to acquire a more activated phenotype after approximately 30 minutes of ischemia [25,26]. In contrast, during coronary bypass operations in adults, less activated cells were observed distal to the ischemic coronary region than proximally [27]. Here, we report the opposite (more activated cells and increased cell numbers leaving the ischemic brain), which may suggest that this is an organ-specific effect.

The most striking finding in this study is the abundance of ‘non-classical’ (CD16^intermediate^ and CD16+) monocytes in venous blood directly after cerebral ischemia. The concentration is much higher than the concentration of cells in the arterial sample, which was drawn simultaneously. The question arises as to where these cells have come from. To systematically address this question, a number of options are possible: 1) these cells have undergone proliferation, 2) the venous sample has a higher viscosity, which increases the concentration of immune cells, and/or 3) cells have migrated from the parenchyma into the intravascular compartment. The first option (proliferation) seems highly unlikely in the short period of half an hour that the increase was observed. The second option, of a temporarily more viscous venous sample, could be an option for example by leakage of plasma into the cerebral parenchyma, or an uptake of fluid by other cells. However, both hematocrit and thrombocyte numbers show stable values and thus argue against this. Therefore, the most valid explanation would be that the cells have migrated into the intravascular compartment, from elsewhere. The cells may have come from the ‘perivascular space’, which is located outside of the endothelial cell layer and inside the parenchymal basal membrane. Histopathologic studies of the brain have revealed that the perivascular space contains macrophages, neutrophils and possibly also lymphocytes [6,28,29]. We hypothesize that the neutrophils and monocytes migrate from their perivascular location, into the vascular space and thereby are increased in the venous sample. The increased permeability of the blood–brain barrier due to the cessation of blood flow may facilitate the migration of cells into the circulation [30-32]. A similar phenomenon has been observed in models of exercise, where within 30 minutes after infusion of adrenaline, amongst others, the ‘nonclassical’ monocytes (CD16^intermediate^ and CD16+) are dramatically increased in the systemic circulation [33]. In this case, the release of these monocytes from the perivascular space is thought to be a direct effect of adrenaline, whereas in the current study, it seems plausible that the ischemia has initiated this process. The phenomenon has also been described in transendothelial models of monocyte trafficking, where the specifically the nonclassical monocyte populations ‘reverse-migrate’ from the tissue into the vascular lumen [34].

The decreased concentrations of neutrophils and monocytes during deep hypothermic CPB may play a role in the above described perivascular phenomenon. As is depicted in Figure 2, neutrophil and monocyte concentrations were much lower than may be expected due to the expansion of the circulating volume by connection to the CPB circuit. Hence, cells may be migrating out of the circulation in response to another, as yet unknown, trigger. At this point, these cells may occupy the perivascular space, only to be released again promptly after cerebral ischemia has occurred.

Regarding the soluble effects of cerebral ischemia, although subtle, significant differences were found after DHCA in IL-6 and sVCAM-1. The higher IL-6 concentration in venous blood after DHCA is presumably a direct effect of ischemia on the endothelium and the intra- and perivascular leukocytes. In line with the activated phenotypes of neutrophils and monocytes, these may rapidly release vast amounts of IL-6. The lower sVCAM-1 concentration may represent the uptake of sVCAM by these cells, as their activated subsets are known to highly express the receptor CD49d [8].

We cannot predict if the observed changes in inflammation are of any clinical relevance. Generally, a more proinflammatory milieu in the first hours of reperfusion predisposes to a more detrimental cerebral outcome [5,35]. However, the difference in inflammation between DHCA and ACP was limited to the time point immediately after the insult, suggesting that the effect of the ischemia was temporary. Of note, this may only be the case in this specific context, as the effect of other inflammatory triggers during surgery (such as the ongoing surgical damage, the use of CPB and cyanosis) are likely overwhelming [36,37].

We acknowledge that this work has important limitations. First, this is a model of cerebral ischemia during deep hypothermia, a temperature not directly applicable to the clinical situation in which adults or children have a stroke. Similarly, although still a matter of debate, neonates may have a different permeability of the blood–brain barrier than adults [38,39]. Second, as dexamethasone was administered to all patients before the start of CPB, we cannot exclude that this may have mitigated the inflammatory response to the ischemia [40]. Finally, due to the many triggers for systemic inflammation that accompany the surgery of these patients, later effects of the ischemia are impossible to tease out. In an animal model, a more ‘clean’ model of cerebral ischemia can be performed, and the evolution of the inflammation can be studied at all desired time points. However, the translation to the human situation is fraught with difficulties, making studies like the current study essential to gain insight into these complex mechanisms.

## Conclusions

In neonates undergoing cardiac surgery at deep hypothermia, we observed an enhanced innate immune response immediately after cerebral ischemia. Most notably, a high number of activated neutrophils and monocytes were found exiting the cerebral circulation. Although the findings are not extreme, this is the first report of such a phenomenon in the very early period after cerebral ischemia and it may hold important clues for the understanding of the immune response to stroke. Further investigation is needed to determine the underlying mechanisms and the further consequences of this rapid immune response, as a better understanding can hold clues for new biomarkers and potentially novel therapeutic options.

## Abbreviations

ACP: Antegrade cerebral perfusion;CPB: Cardiopulmonary bypass;DHCA: Deep hypothermic circulatory arrest;IL: Interleukin;MCP-1: Monocyte chemotactic protein-1;MFI: Median fluorescence intensity;sVCAM-1: Soluble vascular cell adhesion molecule**-**1;TNFα: Tumor necrosis factor alpha

## Competing interests

The authors declare that they have no competing interests.

## Authors’ contributions

SOA, AWLS, FH, NJGJ and BJP were involved in the study design. SOA, FH and FCME enrolled patients and collected the perioperative samples. SOA, KMG and JM performed the laboratory work. SOA, KMG, LK, NJGJ and BJP interpreted the data. All authors read and approved the final manuscript.
